# Recent Progress in Otology

**Published:** 1876-12

**Authors:** J. Orne Green


Recent Progress in Otology.—*By J. Orne Green, M. D.*—*Treatment of Otorrhœa.*—Schwartze replies to Politzer in the next number of the same journal and considers that the failures to relieve in certain cases of otorrhœa treated by the caustic solutions of silver is owing to a want of care in the selection of the time for repeating the cauterization. He considers that the application should be repeated as soon as the slough from the preceeding cauterization has come away. If the interval beteen the applications is too long, no progress is made. He also now uses stronger solutions than formerly, but if a very strong application is indicated he prefers the mitigated solid stick (lapis mitigatus) to the saturated solution. The neutralization with a solution of common salt he still considers advisable from one unfortunate result which oocurred to him years ago, and he also favors neutralization of any of the nitrate which has passed into the nose by salt-water injections into that cavity.

*Exostosis in the External Meatus.*—Aldinger narrates a case of exostosis in both meatuses which gradually filled the passages and produced such inflammation as to require operation. Successful operations in such cases have been so rare that these are of special interest.

The patient, a healthy man thirty-six years old, was first seen with inspissated cerumen in one ear, which had caused deafness. On removal of the small mass it was found that the meatus was largely filled by an exostosis from the upper and posterior wall, which had formed without the patient's knowledge and without any previous disease of the ear. The removal of the cerumen restored the hearing to the normal standard. Examination of the opposite ear showed a similar exostosis.

Eight years after, it was found that the growth in the first ear had closed the passage to a mere slit, which frequently became obstructed from collections of cerumen, and in the course of a few months this slit closed up, shutting in a mass of cerumen between the exostosis and the drum-membrane. The hearing for the voice was completely lost, and the watch was only heard on pressing it against the ear. After this condition had continued for six months suppurative inflammation of the tympanum and meatus set in, and Professor Heinecke removed the growth by chiseling it off in small pieces, while the patient was under chloroform, till a tolerably large passage had been made to the drum-membrane. The reaction from the operation was slight; suppuration set in on the third day, and on the fifth day quite a large splinter of bone came away, and again on the tenth day a still larger piece was removed by the forceps, leaving the meatus about one half its normal size. Examination now showed the drum-membrane in a granular condition, with a small perforation anteriorly. By local treatment this condition was relieved, and the perforation healed, giving a hearing of over three feet for the watch. The seat of the operation cicatrized, but for some months the cicatrix seemed to be again enlarging; eventually, however, the swelling diminished and the patient was left with a fair-sized meatus and perfect hearing.

Six months after the first ear healed the patient had a precisely similar attack of tympanic inflation in the other ear, which was found to be similarly closed by the growth of the exostosis, and after this inflammation had continued for two months the same operation was performed on that side. The result of the operation was equally favorable on this side, a fair meatus being formed, but the tympanum from the long-continued inflammation was more seriously diseased, and a large polypus had formed in it, which required removal. Two months after the operation the tympanic inflammation had subsided, and the perforation of the membrane had healed, giving a hearing of thirty inches for the watch and an equally good hearing for the voice.

In regard to the method of operating, it is only said that narrow chisels were used, that they were applied directly to the base of the exostoses without previous incision of the skin, and that quite strong blows with the hammer were necessary. It was found to be impossible to see, and the operation was done chiefly by feeling.

Gout and syphilis, which have been considered by some authors to be the causes of these exostoses, were absent in this case.

*The Yellow Spot at the End of the Manubrium.*—In looking at the drum-membrane of a healthy ear by means of reflected light, the manubrium of the hammer is seen inserted in the membrane, running downwards and backwards from the upper edge of the tympanic ring and ending at about the centre of the membrane by what appears like and has been described as a spatula-shaped extremity. This spatula-shaped widening of the end of the manubrium appears of a decidedly yellowish color and looks like an opacity of the membrane at that spot. Politzer explained the appearance as an opacity caused by the radiating fibres of the membrane being here heaped upon each other, where they centred on the end of the manubrium, but Trautman from his investigations is led to a different and more scientific explanation, which is also of practical value as it gives another method of determining the position of the hammer and of judging of the condition of the drum-membrane.

From a study of the shape of the hammer, Trautman finds that the manubrium is divided into three edges and three imperfect surfaces. The outer of these edges, that towards the drum-membrane, runs from the short process downwards to near the end of the manubrium, when it bends first somewhat backwards and then forwards; the lower portion of this edge is sharp, the upper more blunt, and as the drum-membrane is attached only to this edge it follows that the attachment of the membrane is narrowest where the edge is sharpest, and this is a little above the spot where the well-known yellow spot is seen. As this edge approaches the very end of the manubrium it divides into two arms, a half-millimetre apart. In the same manner an anterior and a posterior edge can be distinguished, forming three surfaces: an anterior towards the anterior segment of the drum-membrane, an upper or inner towards the promontory, and a posterior towards the posterior segment of the membrane. The anterior lies in its lower third, at an angle of about forty-five degrees with the anterior segment of the drum-membrane, being twisted on the long axis of the hammer, and consequently faces somewhat outwards. Bearing in mind now the angles at which the drum-membrane lies to the axis of the meatus, it will be found that this angle of forty-five degrees at which the anterior surface at its lower third projects from the drum-membrane explains the appearance of the yellow spot seen on inspecting the membrane. In looking through the speculum the outer edge of the manubrium is seen as a yellowish-white, sharply defined line passing downwards from the short process, most distinct about two millimetres above the end of the manubrium where the edge is the sharpest. At the end of the manubrium this edge is seen to bend a little forwards and downwards. The posterior surface of the manubrium can also be seen behind this white line and followed downwards by its indistinct outline from the short process about two thirds of the length of the manubrium; it appears less distinct than the outer edge just described, because it does not lie in direct contact with the membrane ; its color is yellowish-white, with a tint of red derived from the reflection of the promontory, but much less white than the outer edge of the bone; it is from one-half to three-fourths of a millimetre broad at the short process, and gradually narrows till it merges into the outer edge. The lower portion of the anterior surface of the manubrium is seen in the same manner through the membrane and forms the yellow spot anterior to the end of the manubrium.

Trautman confirmed this explanation by taking a fresh preparation and coloring the anterior surface of the manubrium red; he found that the yellow spot thus became red, but no change was produced by coloring any other parts of the tympanum. He also found that by puncturing the yellow spot with a fine needle the anterior surface of the manubrium was always pricked; also that by rotating the hammer on its long axis the size of the yellow spot was increased or diminished according as the anterior surface was exposed or hidden.

The yellow spot is of value for diagnostic purposes, according to Trautman, in the following conditions:

(1.) In thickening of the epidermal layer the yellow spot would disappear earlier than the sharp edge of the manubrium.

(2.) Opacities of the membrane without simultaneous thickening would only alter the color of the yellow spot.

(3.) Rotation of the hammer on its long axis alters the form of the yellow spot.

(4.) If the yellow spot does not move on inflation of the ear, either anchylosis of the malleus and incus has taken place or the malleus is attached to the labyrinth wall.

*Perforation of the Mastoid Process.*—In the last two volumes of the *Archiv fur Ohrenheilkunde* Schwartze continues his record of cases of perforation of the mastoid, which was begun several years ago, and which has now reached fifty in number. The publication is not yet complete and he has not given his conclusions, but some points of practical interest should be mentioned here, as it will be some time before the record is finished. The cases are given in full with the well-known care of the author, and embrace many varieties of mastoid disease and its complications. Begun years ago, before the disease was as well understood as now, the whole article serves to show the gradual development of the pathology of the disease and of the operation of perforation. After the publication is completed we shall have occasion to refer to it again in these reports, but now desire to call attention to one or two points.

In regard to the method of operating: in the earlier cases the trephine and the drill were used for opening the bone, but Schwartze has now given up both of these instruments, and uses instead a sharp gouge and chisel, with which the bone is gradually cut away. He was led to prefer the gouge from his experience in cases of hyperostosis in which the mastoid cells were partially filled with a growth of bone or even wholly obliterated. In operating on one such case with the trephine the inner table of the skull was opened and the lateral sinus exposed, owing to the difficulty of judging of the depth to which the instrument penetrated. Again, in such cases it is often necessary to change the direction of the opening and turn more towards the antrum of the mastoid in order to find the cells which are free from the hyperplasia, a thing dangerous and often impossible to do with a trephine. With the gouge and chisel, however, the bone can be removed bit by bit in any direction desired, and, as Schwartze thinks, there is much less likelihood of injuring the brain.

Among his cases Schwartze has met several instances of complete hyperostosis of the mastoid, all of the cells and even the antrum itself being obliterated by a solid bony growth. One case of reflex epilepsy from disease of the ear, which was cured by perforating the mastoid cells, is of special interest. The only accidents met with in the operation were exposure of the transverse sinus from removal of the inner walls of the mastoid, and opening of the middle fossa of the skull in the same way. The first case did well. The second is interesting as it occurred in trying to reach the antrum mastoideum; the patient died of traumatic meningitis, and the autopsy showed a malformation of the petrous bone which explained the accident.—*Boston Medical Journal.*

